# Pulmonary artery compression by a localized epicardial hematoma in a patient with idiopathic thrombocytopenic purpura after percutaneous coronary intervention: a case report

**DOI:** 10.1186/s12872-016-0378-0

**Published:** 2016-10-28

**Authors:** Satoshi Kawaguchi, Toshiharu Takeuchi, Naoyuki Hasebe

**Affiliations:** Cardiovascular, Respiratory and Neurology Division, Department of Internal Medicine, Asahikawa Medical University, 2-1-1-1 Midorigaoka Higashi, Asahikawa, Hokkaido, 078-8510 Japan

**Keywords:** Cardiac tamponade, Compression syndrome, Coronary artery bypass grafting, Idiopathic thrombocytopenic purpura, Percutaneous coronary intervention

## Abstract

**Background:**

The most common complication of coronary artery perforation, a rare complication of percutaneous coronary intervention (PCI), is hemopericardium with cardiac tamponade. However, localized extra-coronary bleeding can lead to epicardial hematoma, which is a rare phenomenon. We report the case of an unusual delayed presentation of post-PCI hematoma with unrecognized guidewire perforation.

**Case presentation:**

A 70-year-old man with idiopathic thrombocytopenic purpura (ITP) and a history of coronary artery bypass grafting (CABG) underwent PCI. A bare metal stent was implanted in left main coronary artery (LMCA) after balloon dilation. The procedure was performed without any complications, and the patient was discharged 5 days later. However, the patient was unexpectedly admitted by ambulance with cardiogenic shock and new-onset chest pain the next day. Echocardiography did not show any wall motion abnormalities, but a large mass on the right ventricle outflow tract was detected. Contrast-enhanced computed tomography showed a hematoma compressing the main pulmonary artery trunk and the right ventricle. The patient developed sudden cardiopulmonary arrest and cardiopulmonary resuscitation was successful. The patient died during emergent surgical removal of the hematoma. Large, dark red clots between the pulmonary artery trunk and aorta were observed. The suspected origin of the epicardial hematoma was blood oozing from the stent site in LMCA.

**Conclusion:**

This is an unusual case with delayed development of localized hematoma following PCI in the absence of guidewire perforation. Furthermore, this case illustrated the potential of occasional critical complications in patients with impaired blood clotting undergoing PCI.

## Background

Coronary artery perforation is a less frequent complication of percutaneous coronary intervention (PCI) with potentially fatal consequences [1, 2]. Coronary artery perforation causes cardiac tamponade which results from blood extravasation into the pericardial space. Cardiac tamponade has a poor prognosis and is associated with an inpatient mortality rate of 14.8–15.9 % [1, 2]. However, localized forms of extra-coronary bleeding can lead to hematoma formation that can have potentially serious complications as massive epicardial or under-pericardial hematomas may affect hemodynamics. In this report, we present a patient with pulmonary artery compression by a localized hematoma after PCI which led to cardiogenic shock and death despite emergent surgery.

## Case presentation

A 70-year-old male was admitted to our hospital for perioperative cardiac evaluation of abdominal aortic aneurysm. The patient had undergone coronary artery bypass grafting (CABG) for severe stenosis of the left main coronary artery (LMCA) 4 years before, which consisted of the right internal thoracic artery (RITA) to the left anterior descending artery (LAD) and saphenous vein graft (SVG) to the left circumflex artery (LCX). Due to continual growth of the abdominal aortic aneurysm over the years, surgery was indicated. Left coronary angiogram showed 90 % stenosis of the LMCA and total occlusion of both grafts.

**Fig. 1:**
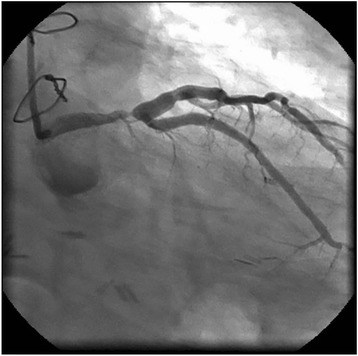
Left coronary angiogram showing severe stenosis of the left main trunk artery

**Fig. 2:**
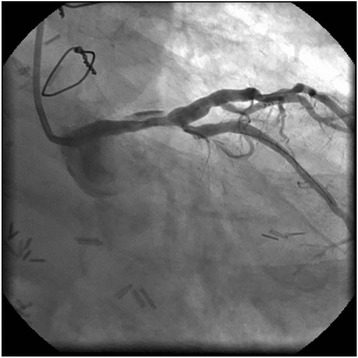
Left coronary artery showing the dissecting lesion after dilation with a 4.0 × 15-mm Quantum Marverick balloon catheter

**Fig. 3:**
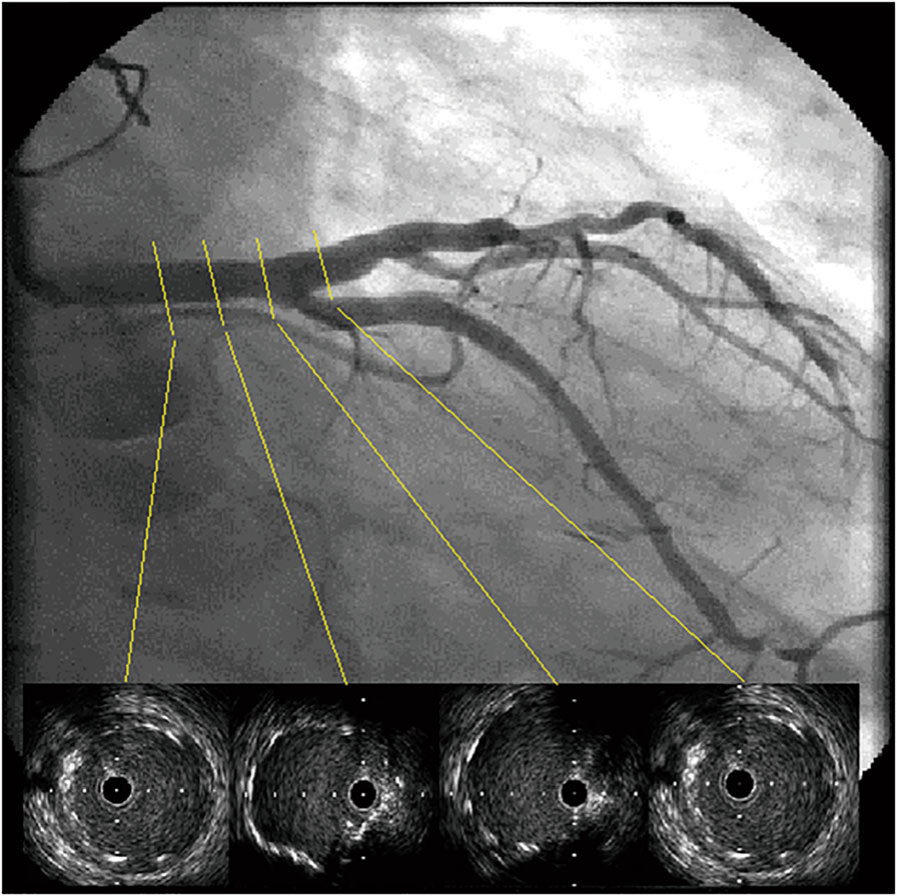
Final angiogram showing no leasions and the bare metal stent fully expanded by intravascular ultrasound. Interventional procedure was succesfully concluded with no complications

PCI was performed for LMCA stenosis (Fig. 1). A 7-Fr sheath was inserted into the right femoral artery, and a CLS4 guiding catheter (Boston Scientific, Natick, MA, USA) was engaged into the left coronary artery (LCA) ostium. A guidewire (SION Blue, Asahi Intecc, Aichi, Japan) was inserted into the distal LAD, and another guidewire (Hi-Torque Pilot 50, Abbott Vascular, Santa Clara, California, USA) was inserted into the distal LCX. Intravascular ultrasound (IVUS) (Intra-focus WR, Terumo Corp, Tokyo, Japan) demonstrated heavy circumferential calcification in the LMCA lesion. After a 4.0 × 15-mm Quantum Maverick balloon catheter (Boston Scientific) was inflated in the lesion, LCA angiogram revealed a large dissection (Fig. 2). A 4.0 × 28-mm Multi-Link Vision stent (Abbott Vascular) was immediately deployed. Angiographic image of the residual dissection disappeared and the stent was dilated by a 5.0 × 15-mm Quantum Maverick balloon catheter. The absence of residual dissection was confirmed by angiography after stent implantation. Next, LMCA-LAD and LMCA-LCX kissing balloon angioplasty was initiated using a 4.0 × 28-mm stent delivery balloon in the LAD and a 3.0 mm × 15-mm Ikazuchi balloon (Kaneka Medics, Tokyo, Japan) in the LCX. The final angiographic imaging showed optimal results, and IVUS imaging of the lesion showed completely sealing by the stent (Fig. 3). A large hematoma at the femoral puncture site and local bleeding was observed after removal of the femoral sheath and manual compression was applied for haemostasis. The hematoma did not appear to have spread the next day and the patient was discharged from the hospital on the fifth day after PCI.

**Fig. 4:**
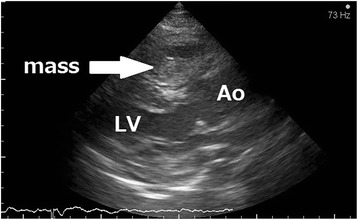
Echocardiography showing the right ventricle compressed by a mass. LV, left ventricle ; Ao, aorta

**Fig. 5:**
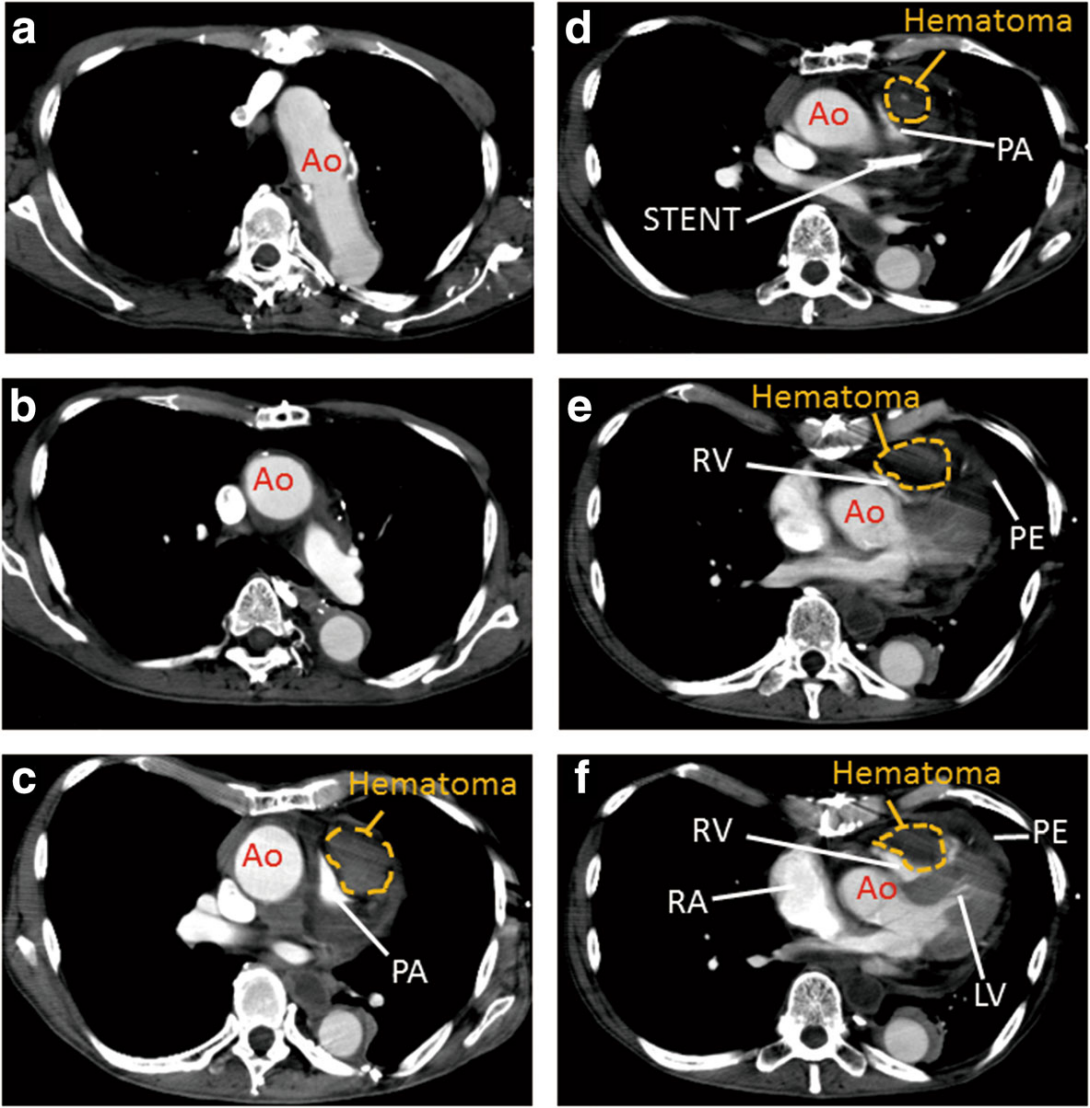
Contrast-enhanced computed tomography showing a massive epicardial hematoma without ascending aortic aneurysm, dissection, or rupture (**a**, **b**, **c**, **d**, **e**, **f**). The hematoma was compressing the pulmonary artery (**c**, **d**) and the right ventricle (**e**, **f**). Ao, aorta ; PA, pulmonary artery ; RA, right atrium ; RV, right ventricle ; LV, left ventricle; PE, pericardial effusion

Unexpectedly, the patient was admitted to our emergency room with new-onset chest pain and dyspnoea the next day after the discharge. His vital signs indicated cardiogenic shock. Electrocardiogram showed ST elevation in leads V1-V3. Although echocardiography could not detect left ventricular asynergy, a large mass on the outflow tract of right ventricular chamber was observed (Fig. 4). Contrast-enhanced computed tomography showed a 40-mm hematoma compressing the main pulmonary artery trunk and the right ventricle (Fig. 5). During examination, the patient suffered sudden cardiopulmonary arrest. He received immediate cardiopulmonary resuscitation and returned to spontaneous circulation after the insertion of a percutaneous cardiopulmonary support device. The patient underwent emergent surgery for the removal of the hematoma after informed consent was obtained from the patient's family. During surgery, large, dark red clots between the pulmonary artery trunk and aorta were observed. The suspected origin of the epicardial hematoma was blood oozing from the LMCA stent site. Despite successful surgical repair, the patient died from aortic rupture induced by external cardiac massage.

## Discussion

Coronary artery perforation is a rare complication of PCI with an incidence of 0.23–0.56 % [1, 2]. However, the most common complication of coronary perforation is the development of haemopericardium with cardiac tamponade. Importantly, recently reported cases of localized epicardial hematoma after PCI have several common features. First, in the majority of cases, hematoma was mainly caused by guidewire perforation during PCI [3–12]. Second, epicardial hematoma after coronary perforation commonly occurs within several hours. Most of the previous reports suggested that compression syndrome by hematomas from cardiac tamponade happened 1 to 5 h after a PCI procedure [3–10]. Third, most patients with localized hematomas had a history of CABG, suggesting that the hematoma was more likely due to the obliteration of the pericardial space by fibrotic adhesions after surgical pericardiectomy.

Although the current patient had also undergone CABG, two other common features in this patient did not match to those of reported cases. In our patient, angiography did not show even minimal extravasation to any extent during PCI by retrospective and inspective observation. Furthermore, the patient presented the first symptom 6 days after PCI, which was a longer-than-expected time for a hematoma to establish when compared with previously reported cases. Chacko et al. reported a patient with SVG perforation during PCI, which resulted in the formation of a localized hematoma that compressed the right atrium 2 days after PCI and caused tachybrady arrhythmia, requiring the implantation of a permanent pacemaker [11]. Maruo et al. reported a case of an extremely delayed pericardial effusion with symptoms of chest pain and anterolateral ST-segment elevation occurring 4 days after coronary intervention [12]. Conversely, there was a possibility that sudden aortic rupture caused the epicardial hematoma; however, this was unlikely in our patient due to two reasons. First, contrast-enhanced computed tomography immediately before the cardiac arrest did not reveal ascending aortic aneurysm, dissection, or rupture (Fig. 5). Second, we observed that the hematoma was dark red and solid, indicating that clots had time to organize over a few days, in contrast to large, bright red and fragile clots that would otherwise be observed in a case of acute aortic rupture leading to hematoma formation with pulmonary artery and right ventricular compression.

The current patient was suffering from ITP for more than 3 years and had a platelet count of 7–8 × 10^4^/μl. Several cases with bleeding complications of PCI in patients with ITP were reported. Fuchi et al. reported a patient with large hematomas around the femoral puncture site after PCI who went into shock [13]. Stouffer et el. reported a patient with ITP who had to discontinue dual antiplatelet therapy due to diffuse petechiae and spontaneous nose bleed 3 weeks after a bare metal stent deployment; the patient continued ITP treatment with 81 mg aspirin per day instead of clopidogrel [14]. Our patient had a tendency to bleed; thus, 100 mg aspirin and 75 mg clopidogrel, both daily, were administered for 3 months prior to PCI. A consultation with the patient’s haematologist was necessary to assess his tendency to bleed and any shift in platelet counts; we decided to perform PCI due to the severe stenosis of LMCA which could lead to sudden death. Although the dissecting lesion in the LMCA was fully covered with the stent, it was highly likely that blood oozing from the dissecting lesion was very slowly pooling in the pericardial space.

Although this critical complication is difficult to predict, it remains a possibility that a hematoma might occur several days after PCI in the absence of guidewire perforation in patients with known bleeding disorders.

## Conclusion

We presented a patient with massive localized epicardial hematoma, which led to the compression of pulmonary artery trunk following PCI. While most pericardial hematoma cases are acute complications by guidewire perforation, pericardial hematoma developed 6 days after PCI in the absence of directly evident complications. Our experience illustrates potential delayed complications after PCI due to bleeding in patients with bleeding dyscrasias.

## Abbreviations

CABG: Coronary artery bypass grafting
ITP: Idiopathic thrombocytopenic purpura
IVUS: Intravascular ultrasound
LAD: Left anterior descending artery
LCA: Left coronary artery
LCX: Left circumflex artery
LMCA: Left main coronary artery
PCI: Percutaneous coronary intervention
RCA: Right coronary artery
RITA: Right internal thoracic artery
SVG: Saphenous vein graft
